# The SOD Mimic, MnTE-2-PyP, Protects from Chronic Fibrosis and Inflammation in Irradiated Normal Pelvic Tissues

**DOI:** 10.3390/antiox6040087

**Published:** 2017-11-06

**Authors:** Shashank Shrishrimal, Elizabeth A. Kosmacek, Arpita Chatterjee, McDonald J. Tyson, Rebecca E. Oberley-Deegan

**Affiliations:** 1 Department of Biochemistry and Molecular Biology, University of Nebraska Medical Center, Omaha, NE 68198, USA; shashank.shrishrimal@unmc.edu (S.S.); elizabeth.kosmacek@unmc.edu (E.A.K.); arpita.chatterjee@unmc.edu (A.C.); 2 Department of Physics & Cancer Research Center, Hampton University, Hampton, VA 23668, USA; john.mcdonald@hamptonu.edu

**Keywords:** MnTE-2-PyP, fibrosis, radiation, fibroblast, T cells, adipose, skin, bladder, NQO1, COL3A1

## Abstract

Pelvic radiation for cancer therapy can damage a variety of normal tissues. In this study, we demonstrate that radiation causes acute changes to pelvic fibroblasts such as the transformation to myofibroblasts and the induction of senescence, which persist months after radiation. The addition of the manganese porphyrin, MnTE-2-PyP, resulted in protection of these acute changes in fibroblasts and this protection persisted months following radiation exposure. Specifically, at two months post-radiation, MnTE-2-PyP inhibited the number of α-smooth muscle actin positive fibroblasts induced by radiation and at six months post-radiation, MnTE-2-PyP significantly reduced collagen deposition (fibrosis) in the skin and bladder tissues of irradiated mice. Radiation also resulted in changes to T cells. At two months post-radiation, there was a reduction of Th1-producing splenocytes, which resulted in reduced Th1:Th2 ratios. MnTE-2-PyP maintained Th1:Th2 ratios similar to unirradiated mice. At six months post-radiation, increased T cells were observed in the adipose tissues. MnTE-2-PyP treatment inhibited this increase. Thus, MnTE-2-PyP treatment maintains normal fibroblast function and T cell immunity months after radiation exposure. We believe that one of the reasons MnTE-2-PyP is a potent radioprotector is due to its protection of multiple cell types from radiation damage.

## 1. Introduction

Radiation-induced fibrosis is a medical condition that develops several months to years after exposure to ionizing radiation. It is characterized by (1) injury to normal tissue, (2) fibroblast activation or myofibroblast formation as reviewed by Straub et al, [1] (3) excessive deposition and decreased degradation of extracellular matrix and (4) in some cases inflammation [1]. Reactive oxygen species (ROS) generated due to radiolysis of water molecules can account for 60–70% of the total acute damage [1,2,3]. Previously, upregulation of nicotinamide adenine dinucleotide phosphate (NADPH) oxidase 4 (NOX4) and other NOX enzymes have been shown to be a secondary source of ROS generation after irradiation [4,5]. This secondary source of ROS generation is sustained due to activation of the transforming growth factor-beta 1 (TGF-β1) signaling pathway, which is known to upregulate NOX4 expression and downregulate antioxidant genes through the activation of transcription factors and epigenetic remodeling in fibroblast cells [4,5,6,7,8]. Furthermore, ROS and TGF-β are also thought responsible for late stage fibrotic symptoms that develop months to years after initial radiation exposure.

The male urogenital region consists of the prostate, bladder, skin, fat, colon, rectum and other reproductive organs. These organs are frequently damaged during radiation therapy for prostate cancer and colorectal cancers. Radiation damage can have acute and chronic effects on normal tissue surrounding the radiation field. Damage to these organs can result in chronic side effects such as rectal bleeding, diarrhea, dysuria, sexual dysfunction, urethral stricture and inflammation. Other acute but transient side effects observed due to radiation damage also include: hair loss, inflammation, low blood counts (myelosuppression), skin rash, sores and ulcers. Currently, there are no therapies approved by the Food and Drug Administration (FDA) to manage or prevent these side effects of radiation therapy.

Radiation is also known to cause the transdifferentiation of different cell types into myofibroblasts. These cells primarily arise from fibroblast cells and are responsible for the production of excessive collagen and other extracellular matrix (ECM) components that lead to formation of the fibrotic tissue. Unrestrained buildup of fibrotic tissue disrupts the normal organ architecture and ultimately leads to organ failure [7]. Macromolecules of the ECM produced by myofibroblasts include α-smooth muscle actin (α-SMA) and an increase in the production of fibrillary collagens (types I, III, V and VI) [5]. Collagen type III alpha 1 chain (COL3A1) is a fibrillar collagen that is known to be expressed in the following tissues of the pelvis: prostate, rectum, testicle, ureter, urethra, urinary bladder, uterus and skin tissue [9]. It is a structural constituent of the extracellular matrix and is known to interact with type I collagen to form fibrotic lesions. Upregulation of COL3A1 is frequently correlated with the fibrotic phenotype of the prostate and an active TGF-β1 signaling pathway [9,10,11]. Moreover, downregulation of COL3A1, using anti-fibrotic agents, has been correlated with the attenuation of the fibrotic phenotype in different organs [9,10,11,12,13,14,15,16]. Therefore, COL3A1 could potentially serve as marker for the fibrotic phenotype in the tissues and organs of the pelvis exposed to radiation.

Fibrosis and abnormal collagen deposition is associated with infiltration of immune cells in the adipose tissue [17,18]. It is suggested that the adipose tissue is sensitive to radiation and may undergo morphological and functional alterations to drive the inflammation mediated fibrotic phenotype [19]. Acute radiation-induced changes include reduction in adipocyte size and increased number of mature adipocytes. Furthermore, the expression of NADPH oxidase was upregulated in parallel with a downregulation of manganese superoxide dismutase (MnSOD) after the irradiation of adipose tissue. This leads to an acute increase in oxidative stress in the adipose tissue after a lethal dose of radiation. However, chronic changes in the adipose tissue following fractionated radiation treatment have not been investigated in the development of fibrosis.

Ionizing radiation can trigger and modulate the inflammatory response by regulating the differentiation of lymphocytes. T lymphocytes are the main players in the cell-mediated adaptive immune response and fibrosis. Naive CD4+ and CD8+ T-helper (Th) cells can differentiate into functional subsets known as Th1, Th2 and Th17 cell types. Differentiation into these cell types governs the balance between pro-inflammatory and anti-inflammatory cytokines. A higher expression of Th2 cytokines and a reduction of Th1 cytokine production has been reported after radiation exposure and other fibrotic disorders. Specifically, Th2 cytokines IL-4 and IL-5 have been found to be upregulated during fibrosis [20,21,22,23,24]. However, Th17 cells do not appear to be affected by radiation exposure. 

Th cell types can affect differentiation of macrophages. Production of the Th2 cytokine, IL-4, can polarize macrophage differentiation into M2 type macrophages [25,26,27]. These polarized M2 macrophages can directly produce and secrete TGF-β1, which can exacerbate the fibrotic process after radiation exposure [26,28]. TGF-β1 produced by the macrophages has also been linked to the production of myofibroblasts via the process of macrophage-myofibroblast transition in a mouse model of unilateral ureteric obstruction [29]. M1 macrophages are also recruited and they help to sustain the immune imbalance by recruiting Th2 cells to the damaged site. Thus, there is an interplay between macrophages, T cells and fibroblasts that promote a fibrotic phenotype.

Manganese (III) Meso-Tetrakis-(*N*-ethylpyridinium-2-yl) (MnTE-2-PyP), is an antioxidant drug that mimics superoxide dismutase (SOD) enzyme activity. This drug acts as a broad mimic of SOD and is present in the mitochondria, nucleus, cytoplasm and extracellular matrix [30]. The addition of MnTE-2-PyP does not affect the native SOD activity levels [31]. MnTE-2-PyP has passed all safety toxicity testing mandated by the FDA [32] and is currently in phase I/phase II clinical trials for treatment of atopic dermatitis. Previously, we have shown that MnTE-2-PyP protects fibroblast cells against radiation-induced damage by inhibiting the TGF-β1 signaling pathway [4] in vitro. MnTE-2-PyP, has been shown to inhibit Th2 cell immune responses in an asthma model [33]. We have also shown that MnTE-2-PyP protects normal prostate tissues from acute radiation-induced damage in rats as a model for prostate cancer radiation therapy [34]. However, there has been no definitive data to show that MnTE-2-PyP protects the development of chronic radiation-induced fibrosis in the urogenital region [4,35,36,37,38,39].

In this study, we have investigated the use of MnTE-2-PyP as an anti-fibrotic modulator of the immune system in response to radiation exposure in chronic animal models. We show in a two-month, post-radiation ex vivo model that fibroblasts isolated from animals irradiated with MnTE-2-PyP treatment were significantly less activated and were significantly less senescent as compared to fibroblasts obtained from mice that had been irradiated alone. Using this same model, the splenocytes and lymphocytes were analyzed and MnTE-2-PyP irradiated mice had higher Th1:Th2 ratio as compared to irradiated alone animals. In the six-month post-radiation model, MnTE-2-PyP treatment significantly protected from collagen deposition in the bladder and the skin of irradiated mice. MnTE-2-PyP treatment also protected from loss of adipocyte size due to radiation exposure and decreased T cell accumulation in the adipocyte tissues.

## 2. Materials and Methods

### 2.1. Cell Lines

Normal human prostate fibroblasts (cat. 4430) were purchased from ScienCell Research Laboratories (Carlsbad, CA, USA). Normal human prostate fibroblasts were cultured in Fibroblast Medium (FM, cat. 2301) on poly-L-lysine coated flasks (2 µg/cm^2^) according to the protocol provided by ScienCell Research Laboratories. Cells were used for experimentation until the population doubling time increased by 50% (approximately one month after recovery from cryopreservation). P3158 cells are normal human prostate cells immortalized by Dr. McDonald J. Tyson. Immortalization was performed by using pBABE-hygro-hTERT plasmid, a gift from Dr. Robert Weinberg (Addgene, plasmid #1773, Cambridge, MA, USA) [40]. These cells were cultured in RPMI-1640 medium, supplemented with 10% fetal bovine serum (FBS) and 1% penicillin/streptomycin.

### 2.2. Animal Husbandry

Six- to eight-week-old, male, C57BL/6 (Charles River Laboratories, Wilmington, MA, USA) mice were used for all experiments. Animals were housed five animals per cage in standard mouse cages in the animal facility at the University of Nebraska Medical Center (UNMC). Mice were exposed to a 12 h light/12 h dark cycle, fed and watered *ad libitum*. All experimental protocols were reviewed and approved by the UNMC Institutional Animal Care and Use Committee (14-054-08-FC).

### 2.3. Animal Radiation Scheme

Six- to eight-week-old, male, C57BL/6 mice were treated 24 h prior to radiation with an i.p. injection of phosphate buffered saline (PBS) or MnTE-2-PyP (10 mg/kg). The following day the mice were anesthetized with ketamine 100 mg/kg/xylazine 5 mg/kg and then placed under lead shielding so that only their lower abdomens were exposed to radiation, using an X-ray box irradiator (Rad Source RS-2000). X-rays were administered ~1 Gy/min. Mice were exposed to 7.5 Gy on five sequential days for a total of 37.5 Gy accumulative dose. During the week of radiation therapy and the week following radiation, the mice were administered either PBS or MnTE-2-PyP (5 mg/kg) three times a week. At two weeks post-radiation, animals were administered PBS or MnTE-2-PyP (10 mg/kg) weekly until the end of the experiment. Animals were sacrificed at two months and six months post-radiation.

### 2.4. Measuring Th1 and Th2 Cytokine Levels from Mouse Spleens and Lymph Nodes

Two months post-radiation, at the time of sacrifice the spleen and lymph nodes (inguinal, lumbar and sacral) were isolated. Single cell suspensions were prepared by pressing the organs through a 70 µm mesh and lysing red blood cells with Ammonium-Chloride-Potassium (ACK) buffer. T cells were plated at a density of 1 × 10^6^ cells/ml in stimulation media [RPMI-1640 medium (Hyclone, cat. SH30027.01, Logan, UT, USA), supplemented with 10% FBS, 2 mM L-glutamine, 25 mM HEPES, 1 mM sodium pyruvate, 1% nonessential amino acids, 55 µM 2-mercaptoethanol, 100 units/mL penicillin/streptomycin for 6 h in the presence of 1 µL/mL Leukocyte Activation Cocktail with GolgiPlug^™^ (BD Bioscience, Carlsbad, CA, USA, cat. 550583). Following stimulation, the cells were fixed and permeabilized using BD Cytofix/Cytoperm^™^ kit (BD Bioscience, cat. 554714, San Jose, CA, USA) according to the manufacturer’s protocol. Cells were then incubated in an antibody mixture containing 1 µg each of the following fluorescent antibodies: CD-PerCP-Cy5.5 (BD Bioscience, cat. 561115), CD8-V450 (BD Bioscience, cat. 560471), IL4-APC (BD Bioscience, cat. 562045) and IFNγ-FITC (BD Bioscience, cat. 562019). After washing, cells were filtered and run on a BD LSRII in the Flow Cytometry Core (UNMC, Omaha, NE, USA). Population analysis of Th1 and Th2 type cytokine expression was performed using BD FACSDiva (v8.0.1) software (BD Biosciences, San Jose, CA, USA).

### 2.5. Primary Prostate Fibroblast Isolation and Culture Conditions

Prostates were collected from C57BL/6 mice at 2 months post-radiation or in 6–8-week-old mice that were untreated. After mincing the prostates, they were digested in 5 mg/mL collagenase I (Thermo Fisher, Waltham, MA, USA, cat. 17100017) for 30 min at 37 °C [41]. Tissue fragments were then cultured for 2–3 weeks in Dulbecco’s Minimal Essential Media (DMEM), supplemented with 10% FBS, 1% penicillin/streptomycin and 1% non-essential amino acids with or without 30 μM MnTE-2-PyP (a gift from Dr. James Crapo, National Jewish Health, Denver, CO, USA). All the cells were fibroblasts after 5 days of culture. Purity of the cells was determined using ERTR7 (Abcam, cat. ab51824, Cambridge, MA, USA), a fibroblast marker and Keratin17 (Cell Signaling cat. 4543, Danvers, MA, USA), an epithelial cell marker. All experiments were repeated in triplicate using primary fibroblasts cells collected from prostates of different mice.

### 2.6. Collagen Contraction Assay

We have followed the same procedure for the collagen contraction assay, which we described previously [4,42]. Briefly, 4 × 10^5^ primary human fibroblast cells were seeded in the presence of PBS or 30 µM of MnTE-2-PyP. The next day, cells were either sham irradiated or irradiated with 2–3 Gy of X-rays. After 48 h of incubation, cells were harvested and embedded in 2 mg/mL collagen discs on low-attachment 24-well plates. In some cases, 4 × 10^5^ primary mouse fibroblasts isolated from 2 months after radiation exposure were seeded on collagen discs. After 8 h of incubation, the collagen discs were imaged and areas were measured using ImageJ (National Institutes of Health, Bethesda, MD, USA).

### 2.7. Senescence Assay

We have described the detailed procedure of senescence staining previously [4,42]. Briefly, 2 × 10^5^ primary human fibroblast cells were seeded in the presence of PBS or 30 µM MnTE-2-PyP followed by either sham radiation or radiation with 3 Gy of X-rays. At 48 h post-radiation, cells were fixed and stained with senescence association beta-galactosidase (SA-β-Gal) staining solution followed by another 24-h incubation. In some cases, 4 × 10^5^ primary mouse fibroblasts isolated from animals 2 months after radiation exposure and were cultured for three weeks and then stained with SA-β-Gal staining solution. Brightfield images were captured with an Olympus IX81 inverted microscope (Olympus America Inc., Melville, NY, USA) and inverted images were analyzed and counted by ImageJ.

### 2.8. Real-Time Quantitative PCR

5 × 10^5^ fibroblast cells were treated with PBS or 30 µM of MnTE-2-PyP. Cells were irradiated with 3 Gy of X-rays (Rad Source RS-2000). At 48 h post irradiation, cells were washed with PBS and collected for RNA isolation. RNA isolation was performed using the Quick-RNA™ MiniPrep Plus kit (Zymo Research, cat. R1057, Irvine, CA, USA) according to manufacturer’s protocol. Isolated RNA was checked for concentration and quality using the Infinite M200 Pro plate reader (Tecan, Mannedorf, Switzerland). One-step, real-time RT-PCR was performed using 40 μg of isolated RNA in a total volume of 20 μL with a master mix from the Power SYBR^®^ Green RNA-to-CT™ 1-Step Kit (Applied Biosystems, Foster City, CA, USA, cat. 4391178). All PCR reactions were performed in a Bio-Rad CFX96 Real Time system thermal cycler. RPLP0 gene was used as the internal control gene for analysis. The primer sequences used in real-time qPCR are as follows: human RPLP0, 5′-GTCCTCGTGGAAGGCCC-3′, 5′-AGGAGAGACAGGGAGCTCAG-3′; human NQO1, 5′-CCTTGTGATATTCCAGAGTGGC-3′, 5′-CCAGGCGTTTCTTCCATCCT-3′; human COL3A1, 5′-CTTCTCTCCAGCCGAGCTTC-3′, 5′-TGTGTTTCGTGCAACCATCC-3′; mouse RPLP0, 5′-GCAGGTGTTTGACAACGGCAG-3′, 5′-GATGATGGAGTGTGGCACCGA-3′; mouse NQO1, 5′-GGTAGCGGCTCCATGTACTC-3′, 5′-CGCAGGATGCCACTCTGAAT-3′; and mouse COL3A1; 5′-TGACTGTCCCACGTAAGCAC-3′, 5′-GAGGGCCATAGCTGAACTGA-3′.

### 2.9. Western Blotting

Cell lysates were prepared using lysis buffer as mentioned in previous publication [4]. Cell lysates (40 μg) were separated by electrophoresis on sodium dodecyl sulfate polyacrylamide gel electrophoresis (SDS/PAGE) gels. Gels were transferred onto nitrocellulose membranes for NQO1 and polyvinylidene difluoride (PVDF) membranes for COL3A1 blotting. Blots were incubated with blocking buffer 5% nonfat dried milk in Tris Buffered Saline with Tween 20. To measure protein loading and transfer, we used Ponceau S staining for nitrocellulose membranes and a reversible protein stain kit for PVDF membranes (Thermo Fisher, cat. 1858784, Waltham, MA, USA). Primary antibody incubation was performed overnight in blocking buffer with anti–NQO1 (1:1000, Novus Biological, cat. NB200-209, Littleton, CO, USA) and COL3A1 (1:10,000, Novus Biological, cat. NB600-549). Bands were detected with anti-rabbit (1:10,000, Thermo Fisher, cat. A24537, Waltham, MA, USA) or anti-mouse (1:8000, Thermo Fisher, cat. A24524) IgG conjugated with horseradish peroxidase. Proteins were visualized using the Pierce ECL Western Blotting Substrate (Thermo Fisher, cat. 80196). All Western blots shown are representative of three independent experiments. Images were scanned and density of bands were quantified using ImageJ. Images were normalized to total protein loaded from Ponceau and PVDF protein staining.

### 2.10. Mouse Tissue Processing

Mouse tissues were harvested immediately following euthanasia, fixed for 48 h in 4% paraformaldehyde at 4 °C and then placed in 70% ethanol. The tissues were embedded in paraffin blocks and 5 µm (bladder and skin) or 7 µm (gonadal fat pad) thick tissue sections were cut and placed on glass slides. Some sections were stained with hematoxylin and eosin while others were left unstained.

### 2.11. Trichrome Staining

Tissue sections were deparaffinized in xylenes and rehydrated in graded alcohols, then rinsed in running deionized water. Tissues were re-fixed in Bouin’s solution for 16 h at room temperature, then rinsed for 10 min in running tap water. Nuclei were stained for 10 min in Weigert’s iron hematoxylin and thoroughly rinsed in running warm tap water for 10 min. Muscle, cytoplasm and keratin were stained in Beibrich Scarlet-Acid Fuchsin (0.9–0.1%) for 5 min and rinsed before differentiating for 15 min in 2.5% phosphomolybdic-2.5% phosphotungstic acid. Finally, collagen was stained for 8 min in 2.5% Analine blue, rinsed and then differentiated for 1 min in 1% acetic acid. Sections were dehydrated using graded alcohols, cleared in xylenes and mounted with Permount (Fisher Scientific, cat. SP15, Hampton, NH, USA).

### 2.12. Quantification for Trichrome Staining

Trichrome stained sections were imaged in brightfield mode, with a 20× objective, on a Leica DM4000 B LED microscope (Leica Microsystems, Wetzler, Germany). Bladders were imaged using 4–5 fields of view to capture the entirety of the muscle area. Using ImageJ software, the muscle region of the image was selected and then thresholding was used to select only red pixels (muscle) or only blue pixels (collagen)—white hues were excluded to eliminate holes in the tissue. The percentage of tissue comprised of collagen was averaged for each animal and the mean per group reported. To measure the collagen density in the skin, each section was imaged over the length of the section requiring six evenly spaced fields of view. The region of interest (the dermis, excluding hair follicles and sweat glands) was selected so that only the area containing collagen was included in the analysis. The image was converted to binary based on analysis of the histogram. The collagen density was calculated as the number of pixels representing collagen divided by the total number of pixels in the region of interest (ROI).

### 2.13. Quantification for Epidermal Thickness

The epidermal layer thickness was quantified using the trichrome staining images. For each image, 25 equally spaced measurements were made along the length of the tissue by drawing a line from the junction of the dermis and epidermis to the edge of the epithelial layer. The pixel value was converted to microns by using a factor of 4.06 pixels/µm. A mean epidermal thickness was calculated for each animal using all six images.

### 2.14. Quantification of Adipocyte Diameter

Adipocyte diameter was quantified using H&E stained tissue sections processed by the core Tissue Science Facility at University of Nebraska Medical Center. Four to six images were analyzed per animal and images were collected from regions of tissue that had not been disturbed (folded or torn) during the staining procedure. ImageJ software was used to process the images for segmentation. First, the image was converted to an 8-bit gray scale and then a rolling ball background subtraction was performed, followed by a median filter to remove noise from the image. The image was then converted to binary using a threshold of pixel values above 249. The resulting borders were over estimated by the high threshold and, thus, were enhanced with the “Dilate” function, which added pixels uniformly at the periphery of the adipocyte. The adipocyte borders were manually labeled with the wand selection tool and using a conversion factor of 4.06 pixels/µm, the areas were calculated and the mean/animal reported.

### 2.15. Immunostaining

Tissue sections were deparaffinized in xylenes and rehydrated in graded alcohols, then rinsed in running deionized water. Antigen retrieval was performed by boiling slides in 10 mM sodium citrate buffer, pH 6.0 for 10 min, followed by a 30 min cool down and a 10 min PBS wash. Endogenous peroxidases were quenched for 5 min in 3% H_2_O_2_ in PBS, followed by a 5 min wash. Next, slides were blocked in 2% goat serum for 30 min followed immediately by a 2 h incubation in primary antibody. Primary antibodies included α-smooth muscle actin (1:100, Abcam, cat. ab5694) and CD3 (1:50, Abcam, cat. ab5690). Negative stain controls were incubated in blocking buffer without primary antibody for 2 h. Following several washes in PBS, the sections were next incubated in biotinylated goat anti-rabbit secondary antibody (1:200, Vector Labs, cat. BA-1000, Burlingame, CA, USA) then washed in PBS again. Peroxidase activity was associated to the biotinylated secondary antibody using the Vector Labs ABC Kit (cat. PK-4000) by incubation for 30 min in ABC buffer. Finally, DAB substrate was applied to detect the proteins of interest (Vector Labs, cat. SK-4100) for 5–7 min until the brown color was visible under a microscope. Slides were counterstained by briefly dipping in Harris hematoxylin (Sigma-Aldrich, cat. HHS16, St. Louis, MO, USA), then dehydrated through graded alcohols, cleared in xylenes and mounted with Permount solution.

For both α-smooth muscle actin in skin and CD3 in fat, six random fields of view were captured with a 20× objective, on a Leica DM4000 B LED microscope (Buffalo Grove, IL, USA). Cells staining a deep brown color were manually counted using the “multi-point” function in ImageJ software. The average cells per field were reported and used for statistical analysis.

### 2.16. Statistical Analysis

All in vitro experiments were conducted independently three or more times. Statistical analyses were performed using GraphPad Prism 6 Software version 6.0.5 for windows. Data are expressed as the mean ± standard deviation (SD). For ex vivo and in vivo experiments, each group consisted of 5–10 animals. Data are reported as the mean ± standard error of the mean (SEM). The statistical significance between different groups was evaluated with 1-way analysis of variance (ANOVA) followed by post-hoc Tukey’s test for multiple comparisons and a *p*-value ≤ 0.05 was considered statistically significant.

## 3. Results

**Figure 1:**
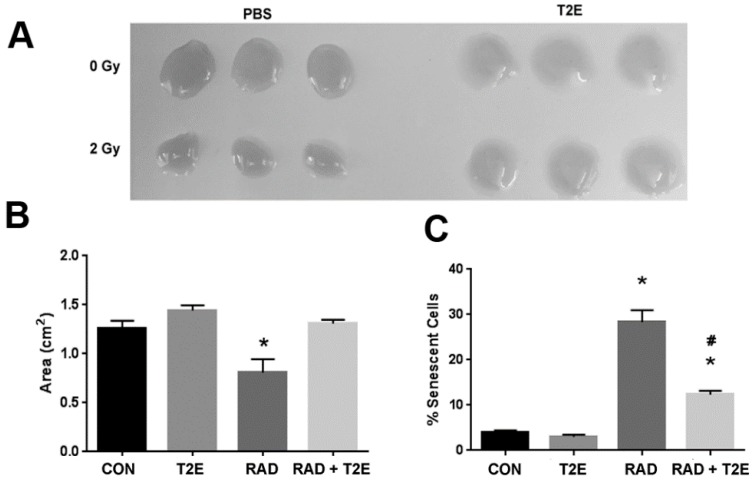
MnTE-2-PyP prevents radiation-induced activation and senescence of human primary fibroblasts. (**A**). Human prostate fibroblasts grown in phosphate buffered saline (PBS) or 30 μM MnTE-2-PyP and then treated with or without 2 Gy of X-rays. After 24 h post-radiation, cells were embedded in rat collagen discs. After 12 h, the collagen discs were measured and the area calculated by ImageJ. (**B**). The average area of the collagen discs was plotted for each group. A 1-way analysis of variance (ANOVA) followed by post hoc Tukey’s test for multiple comparisons was used to determine differences between the disc sizes. (**C**). Human prostate fibroblasts grown in PBS or 30 μM MnTE-2-PyP and treated with or without 2 Gy of X-rays. After 48 h post-radiation, cells were stained for SA-β-gal expression. The number of SA-β-gal expressing cells were quantified by ImageJ using inverted brightfield images. Differences of average percentage of senescent cells were analyzed using a 1-way ANOVA followed by a post hoc Tukey’s test for multiple comparisons. The symbol (*) denotes a significant difference as compared to the control group and the symbol (#) denotes a significant difference when compared to the RAD group. The collagen contraction assays and SA-β-gal staining assays are representative of three independent experiments. CON = control (PBS), T2E = MnTE-2-PyP, RAD = radiation + PBS, RAD + T2E = radiation + MnTE-2-PyP.

Although there has been extensive research showing that MnTE-2-PyP protects normal tissues from radiation exposure, the majority of this work has been conducted using mouse or rat tissues in acute models. We and others have demonstrated that when MnTE-2-PyP is given during radiation, oxidative stress is reduced in normal cells or tissues after radiation [4,34,43]. We wanted to determine whether MnTE-2-PyP protects normal human cells as well. We have previously shown that radiation causes fibroblasts to be activated by inducing their transformation into myofibroblasts and if superoxide is removed during radiotherapy this differentiation is inhibited [4,42]. It is thought that the activated fibroblasts are responsible for fibrosis in normal tissues exposed to radiation [44,45,46]. Therefore, we tested the effect of MnTE-2-PyP on radiation-induced activation of primary human fibroblasts by using the collagen contraction assay. Normal fibroblasts cannot contract their surrounding extracellular matrix; however, due to the acquisition of smooth muscle-like properties a myofibroblast (or activated fibroblast) can contract the surrounding extracellular matrix. Collagen discs were significantly contracted when embedded with the irradiated alone fibroblasts as compared to control fibroblasts (Figure 1A,B). There was no significant difference between the areas of the collagen discs contracted by radiation+MnTE-2-PyP treated cells as compared to control cells. The radiation+MnTE-2-PyP group was significantly different from the irradiated alone group (Figure 1A,B).

Radiation also causes fibroblasts to senescence, which is thought to promote a pro-inflammatory environment in irradiated tissues [47]. To check the capability of MnTE-2-PyP to prevent radiation-induced senescence, we determined the number of senescent primary human prostate fibroblast cells after radiation using senescence associated β-galactosidase (SA-β-Gal) staining. Senescent cells have increased levels of lysosomal β-galactosidase activity as compared to normal cells and this assay is routinely used to detect senescent cells. Radiation significantly increased the percentage of senescent cells (~30%) as compared to controls (Figure 1C). In contrast, MnTE-2-PyP treatment along with radiation greatly inhibited this increase in senescent cells (Figure 1C).

**Figure 2:**
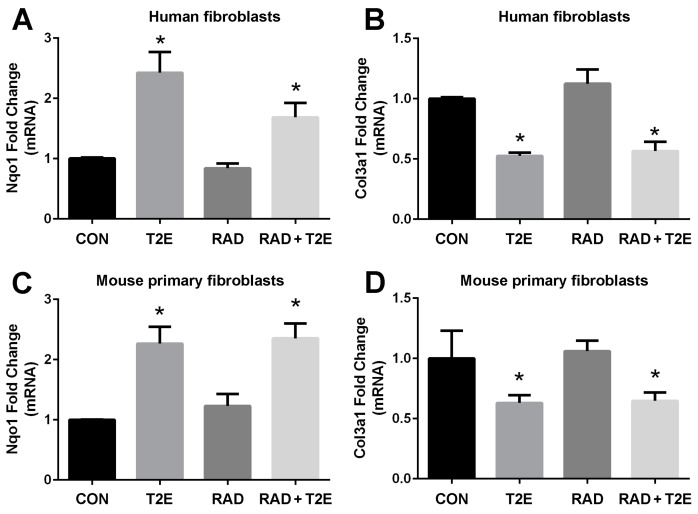
MnTE-2-PyP enhances NQO1 mRNA expression and reduces COL3A1 mRNA expression in human and mouse fibroblasts. (**A**). NQO1 mRNA in human P3158 fibroblasts, which are an immortalized human fibroblast cell line. (**B**). COL3A1 mRNA in human P3158 fibroblasts. (**C**). NQO1 mRNA in primary mouse fibroblasts. (**D**). COL3A1 mRNA in primary mouse fibroblasts. Differences in mRNA expression were analyzed using a 1-way ANOVA followed by a post hoc Tukey’s test for multiple comparisons. The symbol (*) denotes a significant difference as compared to the control group. These data were obtained from 4 independent experiments.

In order to determine the signaling pathways MnTE-2-PyP may be modulating to protect fibroblasts from radiation-induced damage, we conducted RNA sequencing (data not shown). We identified gene expression patterns that were significantly altered with MnTE-2-PyP treatment. We then validated these genes with RT-PCR in both human and mouse fibroblasts. We have found two genes, NQO1 and COL3A1, whose expression levels were consistently altered with MnTE-2-PyP treatment (Figure 2). The NQO1 gene encodes for the NADPH quinone dehydrogenase 1 (NQO1) protein, which is a cytoprotective protein involved in the detoxification of cells when under stress. COL3A1 gene encodes for the collagen 3A1 (COL3A1) protein and this protein is laid down by activated fibroblasts and responsible for inducing fibrosis. NQO1 is normally expressed at low levels and radiation did not affect NQO1 mRNA levels. However, the addition of MnTE-2-PyP produced a significant ~2.5 fold increase in NQO1 mRNA (Figure 2A,C) in the presence or absence of radiation (Figure 2A,C). Conversely, MnTE-2-PyP treatment resulted in a significant reduction (50% reduction) in COL3A1 mRNA with and without radiation in fibroblast cells (Figure 2B,D).

**Figure 3:**
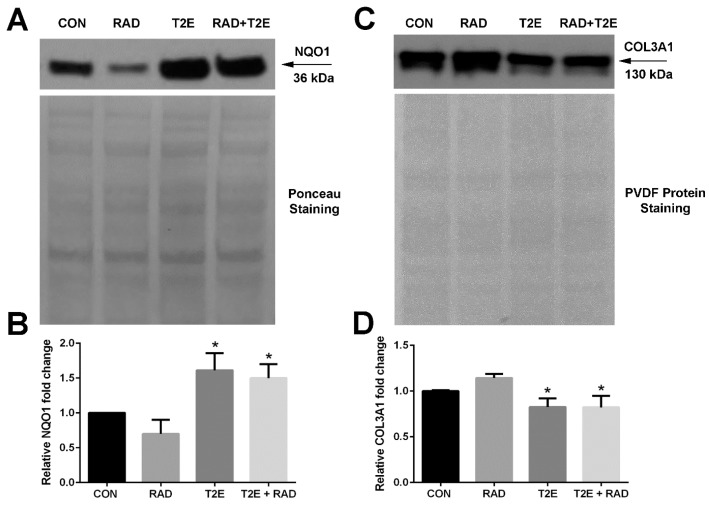
MnTE-2-PyP enhances NQO1 and reduces COL3A1 protein in human fibroblasts. (**A**). Western blot for NQO1 protein with Ponceau staining to control for protein loading. (**B**). Relative densitometry of NQO1 protein levels after normalization to Ponceau staining. (**C**). Western blot for COL3A1 protein with PVDF protein staining to control for protein loading. (**D**). Relative densitometry of COL3A1 protein levels after normalization to PVDF protein staining. Differences in protein expression were analyzed using a 1-way ANOVA followed by a post hoc Tukey’s test for multiple comparisons. The symbol (*) denotes a significant difference as compared to the control group. These data were obtained from 3 independent experiments.

A western blot was then performed for both NQO1 and COL3A1 proteins (Figure 3). We found that MnTE-2-PyP treatment also resulted in a significant ~2-fold increase in NQO1 protein expression (Figure 3A,B). MnTE-2-PyP treatment resulted in a significant decrease in COL3A1 protein expression as compared to irradiation alone; however, this reduction was only ~25%, while the mRNA levels were reduced by 50%.

**Figure 4:**
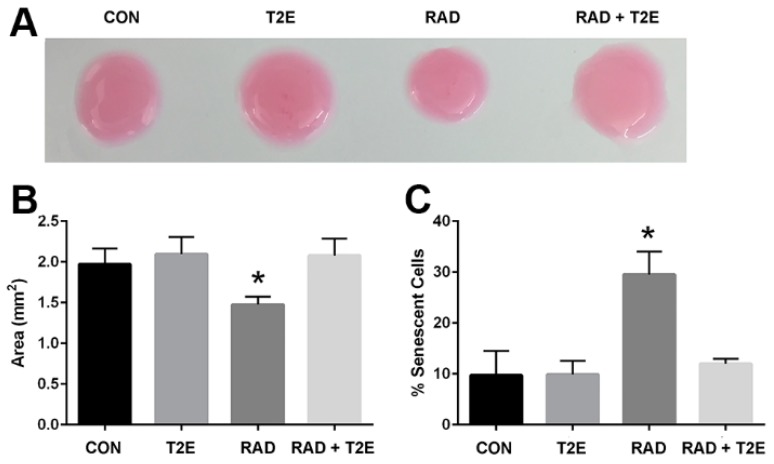
MnTE-2-PyP prevents radiation-induced activation and senescence of mouse primary fibroblasts ex vivo. (**A**). Fibroblasts were obtained from mice treated with +/− MnTE-2-PyP and +/− radiation, at 2 months post-radiation and cultured for two weeks. The cells were then embedded in rat collagen discs for 6 h and then the collagen discs were measured and the area calculated by ImageJ. (**B**). The average area of the collagen discs was plotted for each group. A 1-way ANOVA followed by post hoc Tukey’s test for multiple comparisons was used to determine differences between the disc sizes. (**C**). After 3 weeks of culture, the cells were stained for SA-β-gal activity. The number of SA-β-gal expressing cells were quantified by ImageJ by using inverted brightfield images. Differences of average percentage of senescent cells were analyzed using a 1-way ANOVA followed by a post hoc Tukey’s test for multiple comparisons. The symbol (*) denotes a significant difference as compared to the control group. The collagen contraction assays and SA-β-gal staining assays are representative of 10 mice/group.

We next wanted to determine if these differences produced by radiation that we have observed in our in vitro studies are reflective of in vivo conditions. In these studies, we administered PBS or MnTE-2-PyP with or without radiation (7.5 Gy for 5 consecutive days) and continued to treat the animals with PBS or MnTE-2-PyP weekly until two months post-radiation. We then harvested the prostates from these mice and isolated the prostate fibroblasts. We kept the cells isolated from each animal separate and cultured these cells for two weeks in regular fibroblast growth media without MnTE-2-PyP. We then performed a collagen contraction assay on these cells, which indicates the activation of the fibroblast to a myofibroblast (Figure 4A,B). We found that fibroblasts isolated from irradiated animals could significantly increase the contraction of the collagen disc as indicated by the reduced area of the discs when compared to non-irradiated controls. However, fibroblasts isolated from irradiated mice treated with MnTE-2-PyP were unable to contract the collagen discs (Figure 4A,B). There was no significant difference between fibroblasts isolated from MnTE-2-PyP irradiated animals and non-irradiated controls (Figure 4A,B).

We also investigated the effects of radiation-induced fibroblast senescence in this model. Cells were isolated as described above; however, they were cultured for an additional week. At three weeks of culturing, the cells were stained for β-galactosidase activity (Figure 4C). Fibroblasts from irradiated animals had a 3-fold increase in senescence as compared to fibroblasts isolated from non-irradiated mice (Figure 4C). However, fibroblasts isolated from irradiated mice treated with MnTE-2-PyP displayed no change in the number of senescent cells when compared to non-irradiated control mice (Figure 4C). Thus, the fibroblasts obtained from an animal two months post-irradiation respond in a similar manner as isolated fibroblasts receiving radiation in vitro. These data also indicate that MnTE-2-PyP protects fibroblasts from radiation-induced changes and these protective effects remain months after radiation treatment.

**Figure 5:**
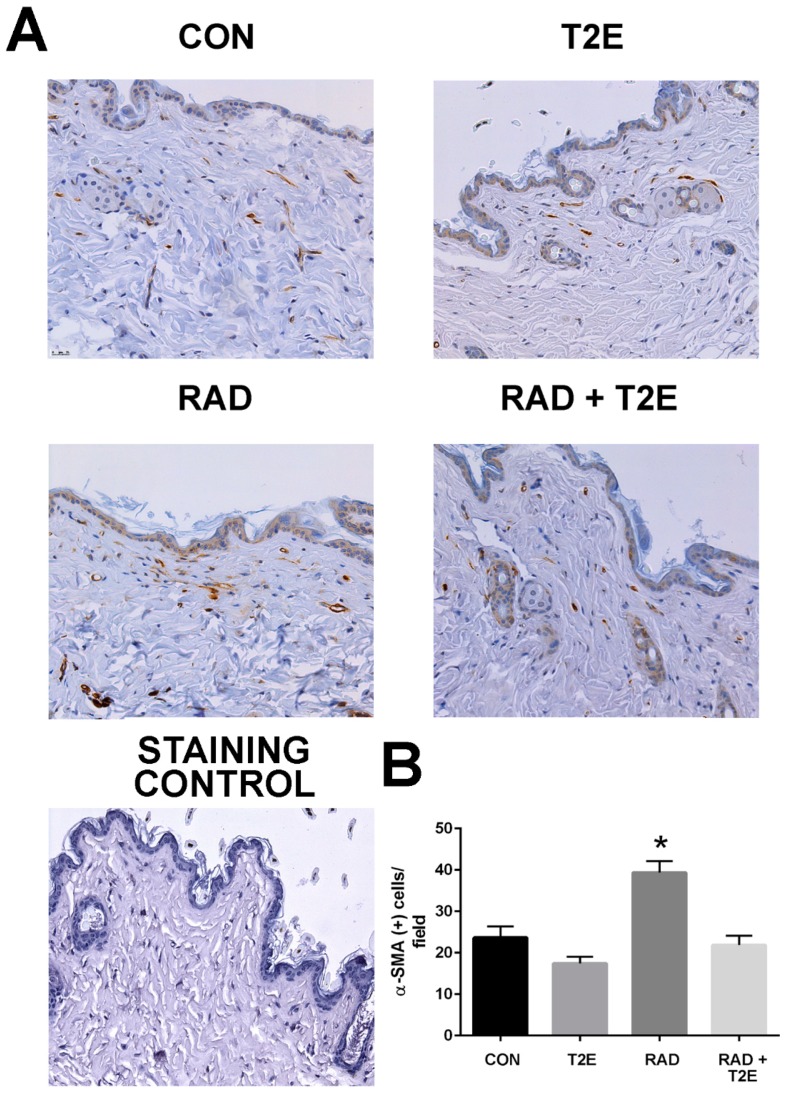
MnTE-2-PyP treatment blocks radiation-mediated upregulation of α-SMA positive cells in skin tissues. Skin sections were obtained from mice treated with +/− MnTE-2-PyP and +/− radiation, at 2 months post-radiation. (**A**). Skin sections were immunostained for α-SMA. Staining control includes entire staining procedure but omits primary antibody. (**B**). The average number of α-SMA positive cells per field for each group. A 1-way ANOVA followed by post hoc Tukey’s test for multiple comparisons was used to determine differences between groups. The symbol (*) denotes a significant difference as compared to the control group. Magnification bar = 25 µm. There were 9–10 animals/group.

At two months post-radiation, whole tissues were collected and processed for immunostaining. In agreement with the findings from the ex vivo fibroblast experiments a significant increase in activated fibroblasts, by alpha smooth muscle actin (α-SMA) staining, was observed in the skin of irradiated mice (Figure 5). However, irradiated mice treated with MnTE-2-PyP had levels of α-SMA in skin tissues similar to unirradiated mice (Figure 5). These data indicate that in vivo, MnTE-2-PyP is blocking the activation of fibroblasts in irradiated tissues.

**Figure 6:**
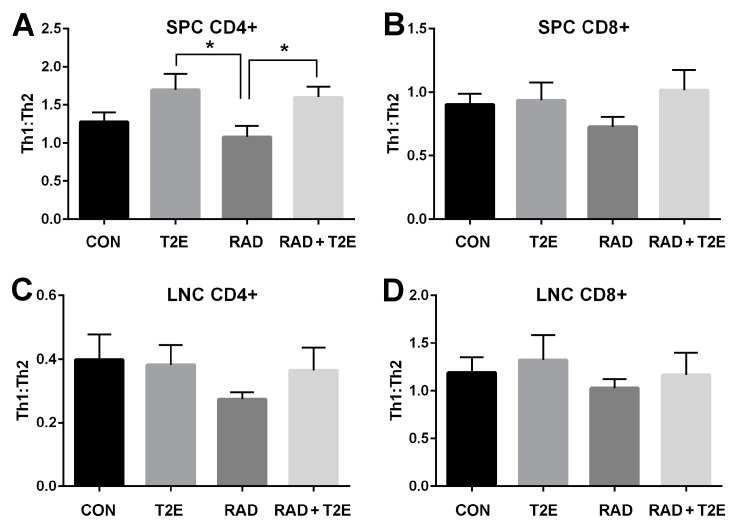
MnTE-2-PyP enhances Th1:Th2 ratio in CD4+ splenocytes 2 months post-irradiation. Splenocytes and lymphocytes were obtained from mice treated with +/− MnTE-2-PyP and +/− radiation, at 2 months post-radiation. These cells were stimulated to enhance cytokine production and then immunolabelled for CD4, CD8, IFNγ (Th1) and IL-4 (Th2). The ratio of Th1: Th2 were calculated for cell type. (**A**). Th1:Th2 in CD4+ splenocytes (SPC). (**B**). Th1:Th2 in CD8 + SPC. (**C**). Th1:Th2 in CD4+ lymphocytes from isolated lymph nodes (LNC). (**D**). Th1:Th2 in CD8+ lymphocytes from isolated from LNC. A 1-way ANOVA followed by post hoc Tukey’s test for multiple comparisons was used to determine differences between groups. The symbol (*) denotes a significant difference. Isolated cells were used from 9–10 animals/group.

Radiation has also been reported to change the immune system. Specifically, T cells have been shown to differentiate less into Th1 type cells and preferentially differentiate into Th2 cells following radiation exposure [48,49,50,51]. MnTE-2-PyP has previously been shown to reduce Th2 cytokine production in an asthma model [33]. Therefore, we investigated the effects of MnTE-2-PyP on T cell differentiation in mice two months post-radiation. The spleens and lymph nodes in the irradiated area were isolated and a single cell suspension was created for each cell type. The cells were stimulated with PMA/Ionomycin/Brefeldin A, fixed, permeabilized and stained for CD4+, CD8+, IFNγ (Th1 cytokine) and IL-4 (Th2 cytokine). The cells were then analyzed by flow cytometry to calculate the cytokine profile for CD4+ and CD8+ cells (Figure 6). Although radiation itself did not induce significant changes in the Th1:Th2 cytokine profile for all cell types investigated, there was a trend indicating that the Th1:Th2 ratio was reduced in mice two months post-radiation (Figure 6). In addition, MnTE-2-PyP treated animals overall displayed an increase in the Th1:Th2 ratios. Specifically, in CD4+ splenocytes, MnTE-2-PyP treatment, with or without radiation, resulted in a significant increase in Th1:Th2 ratios as compared to animals irradiated alone. Similar trends are observed in lymphocytes and CD8+ splenocytes; however, these changes were not significant.

**Figure 7:**
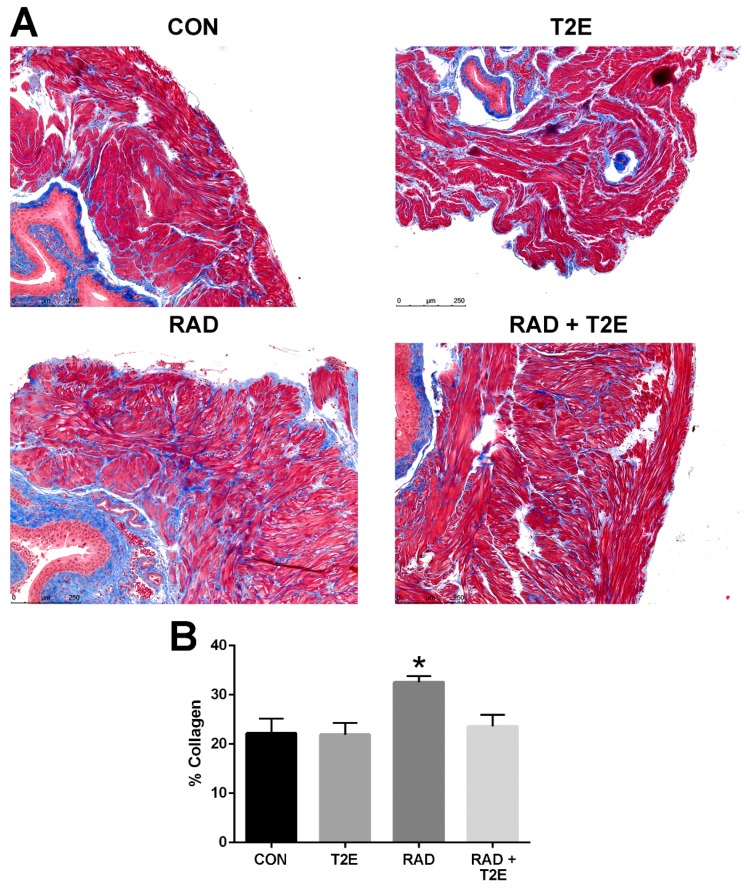
MnTE-2-PyP treatment inhibits fibrosis in bladder tissues at 6 months post-irradiation. (**A**). Representative sections of bladder tissues stained with trichrome to observed collagen deposition from each of the four groups: Control (PBS), MnTE-2-PyP treated alone, Radiation + PBS and Radiation + MnTE-2-PyP. (**B**). The average percent collagen deposition for each group. A 1-way ANOVA followed by post hoc Tukey’s test for multiple comparisons was used to determine differences between groups. The symbol (*) denotes a significant difference as compared to the control group. Magnification bar = 250 µm. There were 5–9 animals/group. Red = cytoplasm, muscle, keratin. Blue = collagen. Black = nuclei.

Tissues harvested two months post-radiation, displayed little immune response and no indication of fibrosis (data not shown). Therefore, we irradiated mice as previously described except that the animals were harvested at six months post-radiation. We collected bladder, skin, adipose, prostate and colon tissues. These tissues were fixed, paraffin embedded and sectioned onto glass slides then stained with Masson’s trichrome to visualize collagen deposition (blue color). Radiation caused a significant increase in collagen deposition in the bladder muscle tissues (Figure 7A,B). Irradiated animals treated with MnTE-2-PyP had significantly less collagen deposition in the bladder muscle tissues and the level of collagen was similar to normal non-irradiated bladders (Figure 7A,B).

**Figure 8:**
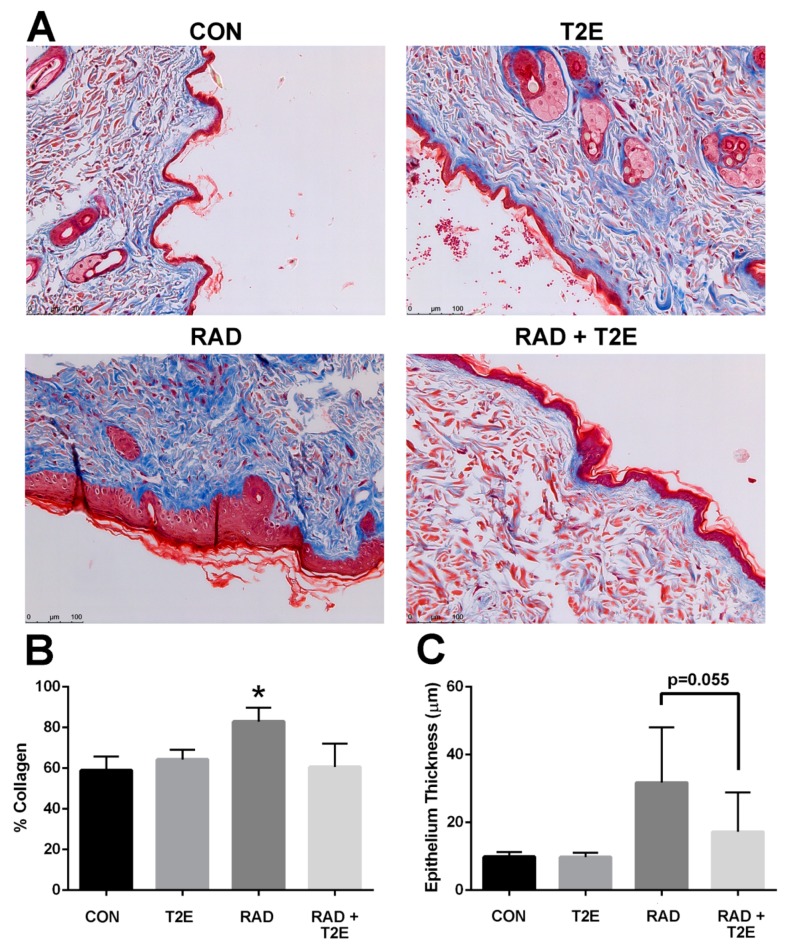
MnTE-2-PyP treatment inhibits fibrosis in skin tissues at 6 months post-irradiation. (**A**). Representative sections of skin tissues stained with trichrome to observed collagen deposition from each of the four groups: Control (PBS), MnTE-2-PyP treated alone, Radiation + PBS and Radiation + MnTE-2-PyP. (**B**). The average percent collagen deposition for each group. (**C**). The average epithelium thickness. A 1-way ANOVA followed by post hoc Tukey’s test for multiple comparisons was used to determine differences between groups. The symbol (*) denotes a significant difference as compared to the control group. Magnification bar = 100 µm. There were 5–9 animals/group. Red = cytoplasm, muscle, keratin. Blue = collagen. Black = nuclei.

Radiation also resulted in significant collagen deposition in the dermis layer of irradiated skin (Figure 8A,B). In some cases, lesions were present in the skin at this time. MnTE-2-PyP treatment resulted in a significant reduction in collagen deposition at 6 months post-irradiation (Figure 8A,B). Radiation also resulted in thickening of the skin epithelium; although, this effect was not uniform across the skin sections and was not significant due to high variation within samples (Figure 8A,C). The MnTE-2-PyP treated irradiated animals showed an overall reduction in epithelial thickening; however, this was not statistically significant. We investigated collagen deposition in the prostate, colon and fat tissues and did not observe any significant differences between groups.

**Figure 9:**
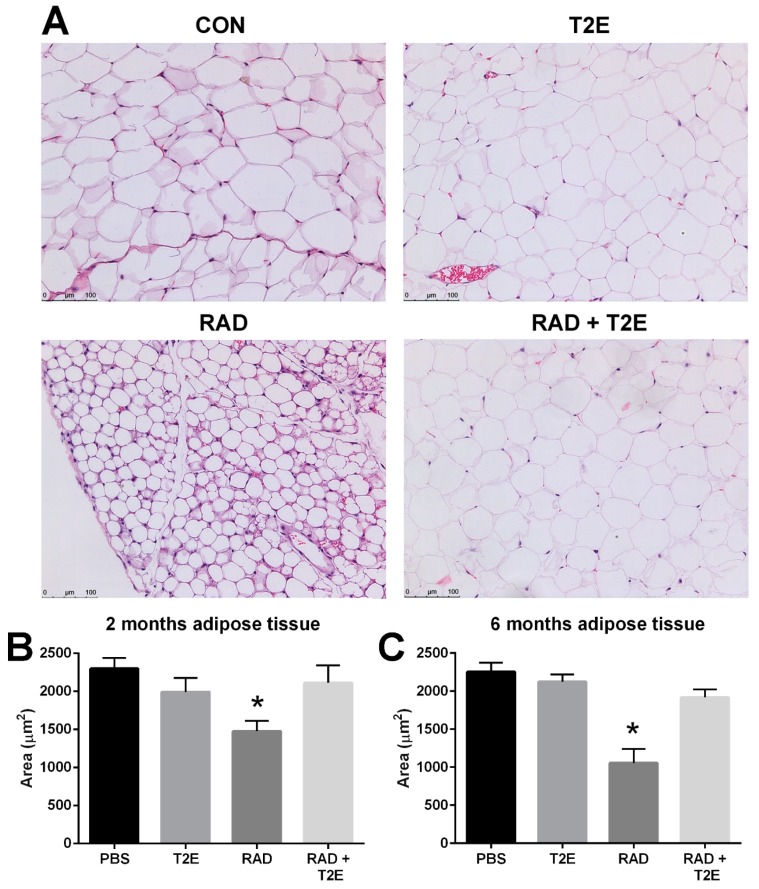
MnTE-2-PyP treatment reverses radiation-induced reduction of adipocyte size at 2 and 6 months post-irradiation. (**A**). Representative sections of adipose tissues at 6 month post-irradiation stained with hematoxylin and eosin to observe adipocyte cell size in each of the four groups: Control (PBS), MnTE-2-PyP treated alone, Radiation + PBS and Radiation + MnTE-2-PyP. (**B**). The average area for adipocyte cells in each group at 2 months post-irradiation. (**C**). The average area for adipocyte cells in each group at 6 months post-irradiation. A 1-way ANOVA followed by post hoc Tukey’s test for multiple comparisons was used to determine differences between groups. The symbol (*) denotes a significant difference as compared to the control group. Magnification bar = 100 µm. There were 5–9 animals/group.

While collagen deposition was not altered in adipose tissues, we did observe radiation-induced changes in adipocyte size in hematoxylin and eosin stained sections at two and six months post-irradiation. Radiation caused a significant decrease in the area of adipocytes and increased the number of adipocytes (Figure 9A–C). MnTE-2-PyP alone showed a slight trend in smaller adipocyte size, although, this was not significant. Irradiated mice treated with MnTE-2-PyP had adipocyte sizes and cell numbers that were indistinguishable from the non-irradiated animals (Figure 9A–C). In addition, at 6 months post-irradiation we also observed that radiation significantly increased the average number of adipocytes per field and MnTE-2-PyP treatment inhibited this increase: PBS (66 cells), MnTE-2-PyP (64 cells), Radiation (150 cells), Radiation+MnTE-2-PyP (71 cells).

**Figure 10:**
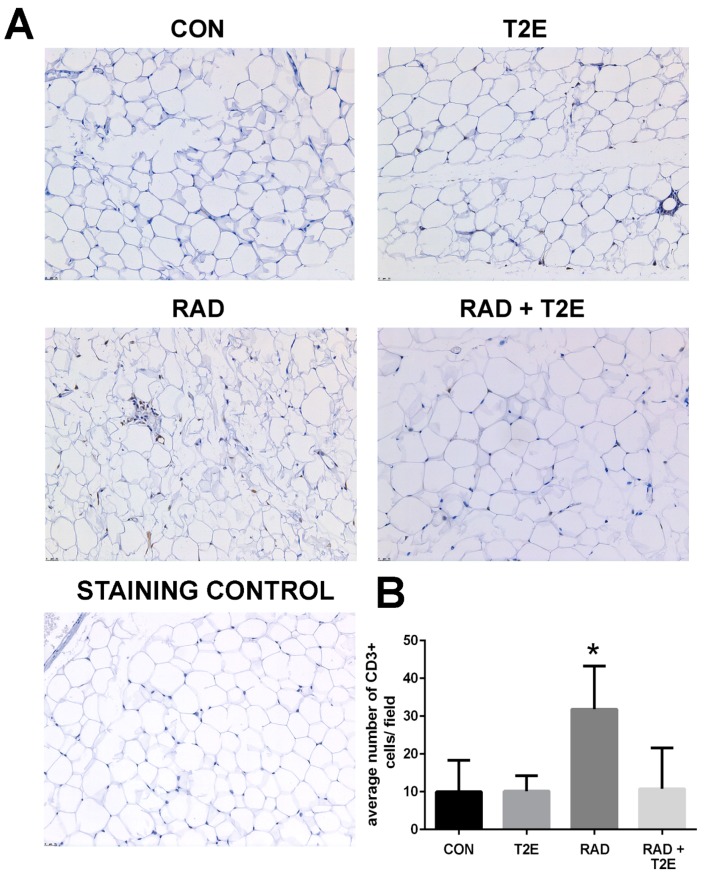
Radiation significantly increases CD3+ cells and MnTE-2-PyP blocks this induction in adipose tissues. (**A**). Adipose sections were obtained from mice treated with +/− MnTE-2-PyP and +/− radiation, at 6 months post-radiation. Adipose sections were immunostained for CD3. Staining control includes entire staining procedure but omits primary antibody. (**B**). The average number of CD3 positive cells per field for each group. A 1-way ANOVA followed by post hoc Tukey’s test for multiple comparisons was used to determine differences between groups. The symbol (*) denotes a significant difference as compared to the control group. Magnification bar = 25 µm. There were 5–9 animals/group.

Because we observed changes in the Th1:Th2 cytokine production in T cells at 2 months post-radiation, we investigated whether inflammation was prevalent in tissues at 6 months post-radiation. From hematoxylin and eosin stained sections we did not observe inflammation in prostate, bowel, bladder and skin tissues. However, we did observe inflammatory infiltrates in irradiated adipose tissues. Adipose tissues were stained for markers of T cells. Radiation caused a significant increase in CD3+ cells, a generic marker for T cells, in adipose tissues (Figure 10). Adipose tissue from irradiated animals treated with MnTE-2-PyP did not display this increase in CD3+ cells (Figure 10). In fact, the CD3+ cell numbers were not significantly different in MnTE-2-PyP irradiated mice as compared to unirradiated animals (Figure 10).

## 4. Discussion

In this present study, we demonstrate that in vitro radiation treatment causes significant morphological changes in fibroblasts, which occur within 48 h after radiation exposure. We translated our in vitro model to show the same radiation-induced fibroblast activation persists for two months and can be detected both ex vivo and in vivo in fibroblasts from an irradiated mouse. Radiation also causes significant changes to T cell immunity. At two months post-radiation, a trend of reduced Th1:Th2 was observed and by 6 months a significant increase in infiltrating T cells was observed in irradiated adipose tissue. MnTE-2-PyP treatment resulted in the reduction of activated fibroblasts and reduction in overall fibrosis of irradiated tissues. The addition of MnTE-2-PyP also produced a higher Th1:Th2 ratio in splenocytes and resulted in the overall reduction of T cells (CD3+ cells) in adipose tissues.

We have previously reported that MnTE-2-PyP and another manganese porphyrin, MnTnBuOE-2-PyP, inhibit fibroblast activation and senescence in both mouse primary prostate and colorectal fibroblasts in vitro [42]. In order to make these findings more clinically relevant, we demonstrated that similar findings were also observed in irradiated primary human prostate fibroblasts treated with MnTE-2-PyP as compared to the mouse fibroblasts.

In addition, we were interested in determining whether MnTE-2-PyP affected gene transcription in the irradiated fibroblasts. We identified two genes, NQO1 and COL3A1 that were significantly affected by MnTE-2-PyP treatment in both human and mouse fibroblasts. MnTE-2-PyP significantly enhanced NQO1 mRNA and protein expression in fibroblasts with and without radiation. NQO1 is a cytoprotective protein involved in detoxification pathways [52,53]. It serves as a quinone reductase, which protects against quinone-induced damage by competing with potentially toxic one-electron pathways [52]. Expression of NQO1 is regulated by the Keap1/Nrf2 pathway, which is dependent on oxidative stress in the cytoplasm for transcriptional activity [52,53,54]. The activity of Keap1/Nrf2 pathway increases in response to oxidative stress and leads to upregulation of antioxidant genes to combat oxidative stress in several fibrosis models [55,56,57,58,59]. Others have also reported that the Nrf2 pathway is enhanced with manganese porphyrin treatment [30,60,61]. We speculate that this increase in NQO1 levels by MnTE-2-PyP primes the cells for a cytotoxic event such as radiation. Thus, NQO1 acts as protective armor to radiation damage by enhancing cytoprotective machinery in the treated cells before the cells are injured by radiation.

We also demonstrated that MnTE-2-PyP treatment resulted in a decrease of COL3A1 mRNA and protein expression in fibroblasts with and without radiation. We had previously shown that MnTE-2-PyP inhibits TGF-β/SMAD signaling pathway in irradiated fibroblasts and COL3A1 is a downstream target of this pathway [4]. It is thought that COL3A1 deposition is in part responsible for fibrosis development [62,63,64]. The reduction of COL3A1 expression may be one way in which MnTE-2-PyP is inhibiting the development of fibrosis.

To further understand if these in vitro changes observed in fibroblasts, 48–72 h after irradiation were relevant in an animal model of irradiation, we performed an ex vivo study. We irradiated the pelvis of mice with fractionated dosing of 7.5 Gy for five consecutive days ± MnTE-2-PyP. At two months post-irradiation, we isolated prostatic fibroblasts and cultured these cells for two to three weeks. At two weeks, some of the cells were embedded in collagen discs to determine if these cells had transformed to a myofibroblast-like cell. An activated fibroblast, or myofibroblast, can contract surrounding matrix while a normal fibroblast cannot. We found that fibroblasts removed from irradiated animals had a more activated phenotype as indicated by increased collagen contraction. At three weeks, a senescence assay was performed on these cells and the fibroblasts from irradiated animals were three times more likely to be senescent than cells from a non-irradiated animal. These findings correlate nicely with the in vitro studies previously performed, indicating that the in vitro model reflects changes in fibroblasts that occur in an irradiated animal model.

We also believe that these findings are profound because the addition of MnTE-2-PyP completely blocked the radiation-induced changes in this ex vivo model. Animals were treated with MnTE-2-PyP three times a week for two weeks following radiation and then once a week until sacrifice. However, when the fibroblasts were isolated these cells were not treated with MnTE-2-PyP for two to three weeks before the collagen contraction or senescence assays were performed. In other words, in the absence of MnTE-2-PyP treatment these cells maintained their inactivated phenotype, suggesting the drug is not masking some underlying permanent damage but has prevented that damage already in vivo. These experiments illustrate that radiation causes sustained changes in these fibroblasts, likely epigenetically reprogramming of the cells, which results in detrimental phenotypic changes of fibroblasts [65]. The addition of MnTE-2-PyP, during and after radiation, appears to protect from this epigenetic remodeling of the fibroblast cells and these fibroblasts remain normal months after radiation and drug treatment. 

In agreement with these in vitro and ex vivo experiments, we also found that irradiated mouse tissues displayed an increase in α-SMA, a marker for an activated fibroblast. Indicating that at two months post-radiation, some of these irradiated fibroblasts have differentiated into activated fibroblasts and are setting the stage for fibrosis to occur in these irradiated tissues. Irradiated animals treated with MnTE-2-PyP did not show an increase in α-SMA positive cells. Again, indicating that MnTE-2-PyP protects fibroblasts from reprogramming into activated fibroblasts in response to radiation.

At the two month time point after pelvic radiation, we did not observe any fibrosis (excessive ECM deposition) in any of the tissues we collected. However, when we repeated these experiments and harvested six months post-radiation, we did observe fibrosis in some of the irradiated tissues. We found excess collagen deposition in the muscle tissues of the irradiated bladder and also observed excess fibrosis in irradiated skin. Again, treatment with MnTE-2-PyP protected both the bladder and skin from radiation-induced fibrosis. We expected to observe fibrosis in the prostate and bowel as well but we did not observe any significant changes at this time. Fibrosis may occur later in these other tissues and if we had waited another couple of months, the fibrosis may have been further developed.

Interestingly, we did see quite significant changes in adipose tissues following pelvic irradiation. Adipose tissues are often overlooked in the context of radiation damage. At two and six months post-radiation, the adipose tissues underwent drastic morphological changes in response to radiation therapy. The average areas of the adipocytes were significantly smaller in irradiated adipose tissues as compared to unirradiated tissues. At two months post-radiation, we observed some inflammation in the irradiated adipose tissues but it was not significant (data not shown). At six months post-radiation, we did observe a significant increase in T cell infiltration into adipose tissues. MnTE-2-PyP treatment significantly prevented the decreased adipocyte size as well as the inflammation in adipose tissues caused by radiation. Others have reported that radiation results in the reduction of adipocyte size after radiation [19]. Poglio et al. suggest that these morphological changes in adipocytes indicates a shift in metabolism. Specifically, radiation may induce mobilization of energy sources for adipose tissues to repair radiation damages.

At two months post-radiation, we also observed changes in the T cell populations. We observed a trend of decreased Th1:Th2 ratios in the splenocytes of irradiated mice, although, it was not quite significant. MnTE-2-PyP treatment resulted in significantly higher Th1:Th2 ratios in irradiated splenocytes as compared to radiated alone cells. We tried to measure these T cell population changes after six months post-radiation but we had some technical difficulties. The antibody for IL-4 had lost its fluorescence and so when we analyzed the cell populations we detected little to no IL-4 production in these splenocytes. This is something that we would like to redo in the future to determine if these changes in T cell immunity persist 6 months after radiation. This switch in more Th2 secreting cells can activate M2 macrophages, which can make TGF-β and promote fibrosis. IL-4 secretion from the T cells can also directly activate the fibroblasts [66,67,68]. Therefore, we believe that this increased inflammation in the adipose tissues may be contributing to the fibrosis of other irradiated tissues.

In conclusion, we have shown that the addition of MnTE-2-PyP to irradiated animals protects from fibroblast activation, fibrosis and inflammation associated with radiation exposure in both acute and chronic radiation models. We have also demonstrated that MnTE-2-PyP protects the adipose tissues from radiation-induced changes and inhibits inflammation in the adipose tissues in chronic models of radiation. We believe that MnTE-2-PyP is a potent radioprotector because this drug protects many cell types from radiation damage acutely and chronically after radiation exposure.
